# Predicting the risk of chemotherapy-induced myelosuppression in clinical practice: an interpretable machine learning approach based on routine laboratory data

**DOI:** 10.3389/fonc.2026.1780678

**Published:** 2026-10-14

**Authors:** Hao Fan, Xuejiao Wu, Zhenyu Cheng, Xiubai Tang, Hui Huang, Keyan Chen

**Affiliations:** 1 Department of Pharmacy, Northeast Yunnan Central Hospital, Zhaotong, China; 2 Department of Pharmacy, Yiliang County People’s Hospital, Zhaotong, China

**Keywords:** chemotherapy-induced myelosuppression, machine learning, prediction model, risk factors, SHAP analysis

## Abstract

**Objective:**

Chemotherapy-induced myelosuppression (CIM) is the most common adverse reaction of tumor chemotherapy and may lead to treatment delay, dose reduction, life-threatening infection, and poor prognosis. Current predictive methods remain suboptimal, with insufficient interpretability and limited clinical application. This study aimed to develop and validate an interpretable machine learning model for early CIM risk prediction using routine laboratory data.

**Methods:**

A total of 1,000 cancer patients from Northeast Yunnan Central Hospital between January 2024 and January 2025 were enrolled and divided into a case group (n=500, with myelosuppression) and a control group (n=500, without myelosuppression).All observed samples were randomly split into a training set (70%) and a validation set (30%). The Least Absolute Shrinkage and Selection Operator (LASSO) was used to select optimal variables for model construction. The predictive performance of 15 different ML models was evaluated using sensitivity, specificity, accuracy, positive predictive value (PPV), negative predictive value (NPV), F1-score, Youden’s index, area under the receiver operating characteristic curve (AUC), decision curve analysis (DCA), and residual boxplots. The best-performing model was selected for SHAP-based interpretability analysis and internally validated using a confusion matrix, clinical impact curve, and calibration curve.Additionally, 488 patients treated at Northeast Yunnan Provincial Central Hospital between June 2025 and November 2025 were selected as an independent external validation cohort for external validation.

**Results:**

After screening, 15 clinical indicators were used for ML model development. All models demonstrated excellent discrimination. The LightGBM model showed the best overall performance (AUC: 0.999 [0.998–1.000]; sensitivity: 0.986; specificity: 0.989; accuracy: 0.987; PPV: 0.989; NPV: 0.986; F1-score: 0.987; Youden’s index: 0.974). According to the LightGBM model, CIM risk increased when the following feature thresholds were met: white blood cell count <5×10^9^/L, mean hemoglobin concentration <325 g/L, monocyte percentage (MONO%) >10%, red blood cell count <4×10^12^/L, mean platelet volume <8 fL, and plateletcrit <0.1%.In the independent external validation (n=488), the LightGBM model remained stable and reliable, with an AUC of 0.940 (95% CI: 0.920–0.959), sensitivity of 0.818, specificity of 0.888, accuracy of 85.2%, and favorable calibration and clinical net benefit.

**Conclusion:**

The LightGBM prediction model developed in this study can enable early identification of high-risk patients, providing a reference for healthcare providers to implement early interventions.

## Introduction

In 2020, approximately 19.29 million new cancer cases and 9.96 million cancer-related deaths were reported globally (1). As a major global public health issue, malignant tumors have become the leading cause of death among residents in China (2). The disease not only imposes severe physical and psychological burdens on patients (3) and diminishes their quality of life (4), but also creates substantial economic strain on families and society (5). Chemotherapy, one of the primary treatment modalities for cancer, exerts therapeutic effects while also inducing various adverse reactions (6). Among these, myelosuppression is the most common and serious chemotherapy-related side effect (7, 8). Myelosuppression refers to reduced activity of hematopoietic precursor cells in the bone marrow, and is clinically diagnosed based on the most severely affected indicator among post-chemotherapy white blood cell,platelet counts, or hemoglobin levels (9). Despite continuous advancements in oncology and the emergence of novel therapeutic agents, chemotherapy remains a cornerstone of systemic cancer treatment. Notably, chemotherapy-induced myelosuppression (CIM) represents the most common dose-limiting toxicity of chemotherapy. It can lead to severe complications including anemia, bleeding, and life-threatening infections. Many patients are forced to undergo chemotherapy dose reduction or even premature treatment discontinuation, which not only prolongs hospital stay but also compromises the overall therapeutic efficacy (10). Therefore, enhancing the prevention and management of myelosuppression holds significant clinical importance.

The clinical symptoms of CIM often exhibit a delayed manifestation. Due to limitations in hospitalization duration, myelosuppression in many patients is only detected after discharge (11). The incidence of myelosuppression induced by cancer treatment is high, and it can easily trigger severe complications such as febrile neutropenia. This may lead to chemotherapy dose reduction, treatment delays, and in serious cases, accelerated disease progression, adversely affecting patient prognosis. These consequences not only increase the healthcare burden but also compromise the anti-tumor efficacy of chemotherapy and reduce patients’ quality of life (12). Therefore, utilizing machine learning models to predict the risk of myelosuppression holds significant clinical value. Research on various risk factors for CIM began relatively early, but most previous studies have been retrospective and observational in nature (13). Most existing CIM risk prediction models focus on a single dimension, such as white blood cell or neutrophil levels. However, current research is largely confined to investigating the relationship between individual predictive indicators and the occurrence of myelosuppression (14, 15), often characterized by limited sample sizes, simplistic model-building methodologies, and overall suboptimal quality. Furthermore, there is a lack of studies comparing the clinical utility of different prediction models, as well as a comprehensive evaluation of various risk prediction models.

Machine learning (ML) algorithms possess the capability to rapidly process complex and unstructured data, and can automatically learn intricate non-linear relationships and interactions. In recent years, they have been widely applied in clinical practice, demonstrating significant advantages in disease prediction and diagnosis (16), and can provide a more precise approach for CIM risk assessment. Therefore, this study aims to develop a risk prediction model for chemotherapy-induced myelosuppression based on machine learning algorithms. By comparing the performance of 15 common machine learning models, and utilizing the Shapley Additive Explanations (SHAP) algorithm to visualize and analyze the overall decision logic of the optimal model, this research seeks to provide a reference for healthcare professionals to rapidly assess and identify high-risk patients for post-chemotherapy myelosuppression, enabling timely and targeted interventions.

## Methods

### Study design

This retrospective cohort study enrolled patients with malignant tumors who received their first chemotherapy at Northeast Yunnan Central Hospital between January 2024 and January 2025. All analyses were performed at the patient level, not at the hospitalization or chemotherapy cycle level. Each patient was included only once, based on their first chemotherapy cycle; multiple hospitalizations or repeated chemotherapy cycles from the same individual were not included to avoid data clustering and information leakage. Patients who developed myelosuppression during hospitalization were assigned to the case group (n=500), while those who did not experience myelosuppression were assigned to the control group (n=500). Additionally, 488 patients treated at the same hospital between June 2025 and November 2025 were selected as an independent external validation cohort.

Additionally, 488 patients treated at the same hospital between June 2025 and November 2025 were selected as an independent external validation cohort (experimental group: n=247; control group: n=241). The study was approved by the Ethics Committee of Northeast Yunnan Central Hospital (Approval No.: kj2025042). As this study involved a retrospective analysis of de-identified and anonymized data, a waiver of written informed consent was granted by the ethics committee. The reporting of this observational study followed the STROBE (Strengthening the Reporting of Observational Studies in Epidemiology) guidelines.

### Study subjects

The diagnostic criteria for CIM were based on the Expert Consensus on Tumor Therapy-Related Myelosuppression (17).

#### Inclusion criteria

(1) Patients diagnosed with malignant tumors according to established diagnostic standards and receiving chemotherapy; (2) Patients who voluntarily underwent chemotherapy with complete medical record data; (3) Patients without anemia, HIV co-infection, or other myelosuppression-related conditions prior to chemotherapy. (4) Age ≥18 years.

#### Exclusion criteria

(1) Patients with severe systemic infections, uncontrolled unexplained infections, or serious hematological diseases (leukemia, lymphoma, multiple myeloma, myelodysplastic syndrome, aplastic anemia, or congenital cytopenia); (2) Patients with pre-existing myelosuppression before chemotherapy or those prophylactically administered leukocyte-elevating drugs; (3) Patients who had used glucocorticoids or glucocorticoid inhibitors within three months prior to chemotherapy; (4) Patients receiving other treatments (eg, immunotherapy), those who discontinued or abandoned chemotherapy, or those with incomplete medical records.

### Clinical endpoint and definition of myelosuppression

The primary endpoint was chemotherapy-induced myelosuppression (CIM), which was defined strictly according to the Common Terminology Criteria for Adverse Events (CTCAE) Version 5.0.

In this study, CIM was designated as grade ≥3 hematological toxicity, a composite endpoint including neutropenia, thrombocytopenia, or anemia. The laboratory threshold criteria based on CTCAE 5.0 were as follows: grade 3 neutropenia (absolute neutrophil count, ANC: 0.5–0.99×10^9^/L), grade 3 thrombocytopenia (platelet count: 25–49×10^9^/L), and grade 3 anemia (hemoglobin: 60–79 g/L); grade 4 severity was defined as ANC <0.5×10^9^/L, platelet count <25×10^9^/L, and hemoglobin <60 g/L, respectively.

Endpoint assessment was performed based on routine blood routine examination on days 7 to 14 after chemotherapy administration, and the nadir hematological value within the current chemotherapy cycle was adopted to determine the occurrence and grade of myelosuppression. Patients without grade ≥3 hematological toxicity were classified into the non-myelosuppression group.

### Sample size calculation

According to China’s first large-scale cross-sectional survey on chemotherapy-induced myelosuppression and clinical management—Survey Report on Chemotherapy-Induced Myelosuppression and Clinical Management Status in China—the overall incidence of myelosuppression following chemotherapy was 44.2%where α = 0.05, δ = 0.05, Z = 1.96, and P = 0.442, the calculated minimum required sample size was approximately 379 cases. In this study, the case group comprised 500 patients and the total sample size was 1000, which adequately meets the statistical requirements of the study.

### Data preprocessing

General demographic data (n = 2) and laboratory indicators (n = 50) were retrospectively extracted from electronic medical records at baseline before chemotherapy, i.e., within 24 hours of initial hospital admission and prior to the first cycle of chemotherapy. All predictor variables strictly adopted pre-treatment baseline values only; no laboratory results obtained after chemotherapy initiation, during the hematological nadir, or at subsequent re-examinations were included in the model analysis. Repeated laboratory measurements during hospitalization were not incorporated to avoid temporal information leakage.

Collected variables included sex, age, routine blood parameters, blood lipids, electrolytes, liver function, renal function, and coagulation profiles. For variables with missing values, raw data were first cross-verified and supplemented via the electronic medical record system. Variables with a final missing rate >10% were directly excluded from analysis, whereas those with a missing rate ≤10% were processed using multiple imputation based on the Random Forest (RF) algorithm.For variables with missing values, we first manually retrieved and supplemented raw data through the electronic medical record system. Considering that this study was based on retrospective real-world clinical data, missing laboratory indicators were mainly caused by routine clinical examination arrangements rather than random omission. Therefore, we did not further conduct formal missing mechanism tests.

### Development and interpretability analysis of the ML model for CIM prediction

The entire dataset was randomly divided into a training set (70%) and a validation set (30%) strictly at the patient level to eliminate information leakage. To create a balanced dataset, the sampling process maintained a 1:1 ratio between the case and control groups within both sets.thereby mitigating potential bias caused by class imbalance. Ten-fold cross-validation and hyperparameter tuning were employed to reduce the risk of model overfitting, with a grid search utilized to optimize model parameter configurations.The Least Absolute Shrinkage and Selection Operator (LASSO) regression was subsequently applied only on the training set to screen and determine the optimal variables.

Fifteen distinct machine learning algorithms were trained and evaluated on the training and validation sets, including: Random Forest (RF), Gradient Boosting (GraBoost), Support Vector Machine (SVM), Logistic Regression (LR), k-Nearest Neighbors (k-NN), Partial Least Squares (PLS), Boosting (Boost), Neural Networks (NNet), Bayesian Methods (Bayes), Linear Discriminant Analysis (LDA), Least Absolute Shrinkage and Selection Operator (LASSO), Adaptive Boosting (AdaBoost), Extreme Gradient Boosting (XGBoost), Categorical Boosting (CatBoost), and Light Gradient Boosting Machine (LightGBM). Model performance was assessed using the following metrics: Receiver Operating Characteristic (ROC) curve, Area Under the Curve (AUC), Sensitivity, Specificity, Positive Predictive Value (PPV), Negative Predictive Value (NPV), Accuracy, F1-Score, and Youden’s Index. Additionally, Decision Curve Analysis (DCA) and boxplots of absolute residuals were used for further evaluation.

The diagnostic efficacy and reliability of the optimal model were further assessed using a Confusion Matrix (CM), Calibration Curve (CC), and Clinical Impact Curve (CIC). The model demonstrating superior performance across these comprehensive metrics was identified as the final optimal model. To facilitate a clearer understanding of the optimal model’s decision-making process, SHAP visualization analysis was conducted. This analysis provided a global explanation of how the most important features contributed to the model’s predictions, quantified the impact of these key features on the predicted CIM probability for individual patients, and interpreted the significant contributions of the predictive features.

### Statistical analysis

All statistical analyses and visualizations in this study were performed using R software (version 4.5.2) and IBM SPSS Statistics (version 27). Continuous data conforming to a normal distribution are presented as mean ± standard deviation (SD), while non-normally distributed data are presented as median [interquartile range, IQR] and were compared using the Mann-Whitney U test. Categorical data are expressed as percentages and were compared using the Chi-square test or Fisher’s exact test, as appropriate. A two-tailed P-value of less than 0.05 was considered statistically significant.

## Results

### Development process of the ML model for CIM prediction

The development workflow of the machine learning (ML) model for predicting CIM is illustrated in **Figure 1**. This study evaluated the computational time of each machine-learning algorithm, assessed the inference speed and performance of each algorithm, and performed hyperparameter tuning for the 15 algorithmic models via grid search. The computational time of each algorithm and tuning results are presented in **Supplementary Table S1**.

**Figure 1:**
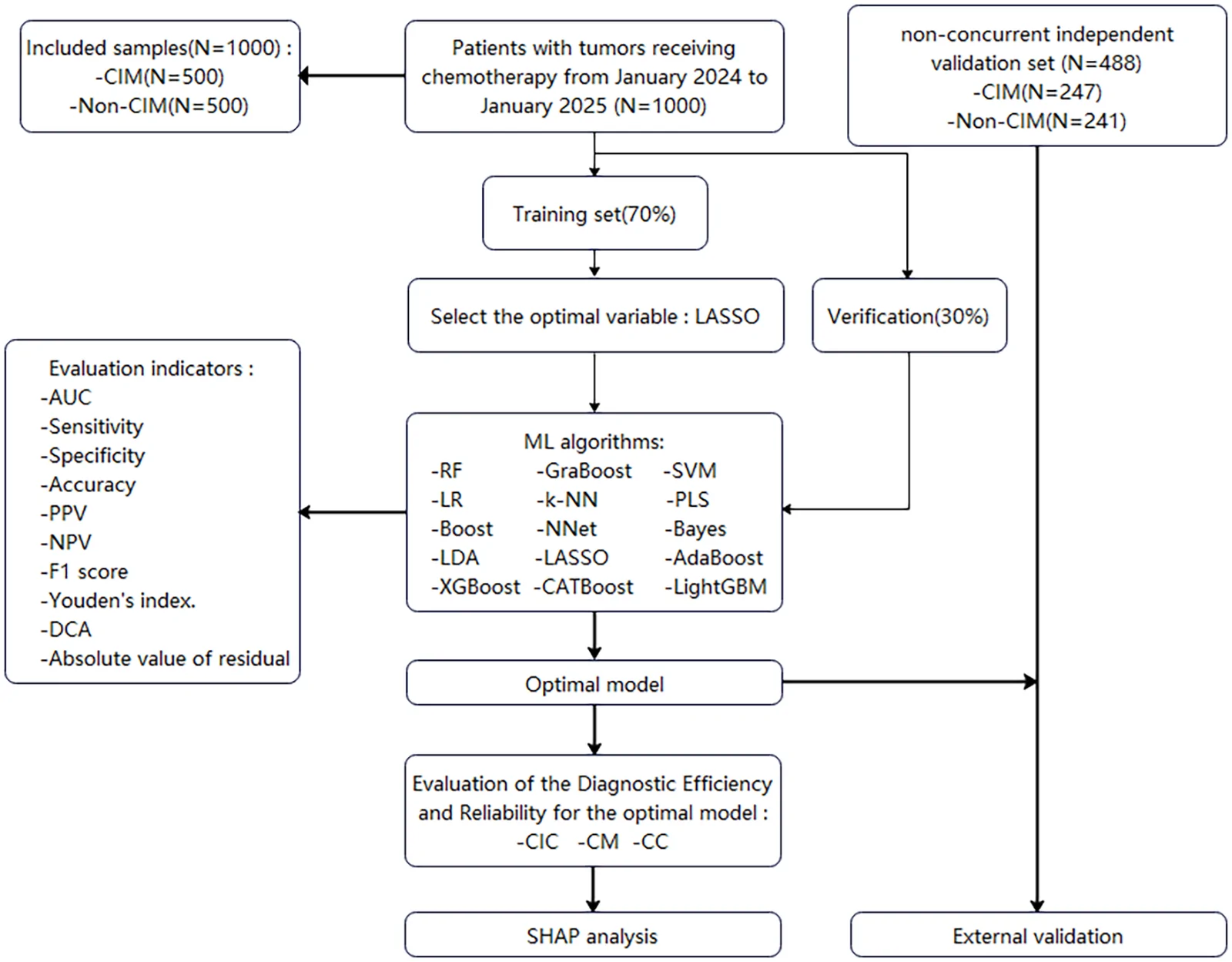
ML development process for CIM.

### Baseline characteristics of the study cohort

Through the establishment of a balanced cohort, this study included a total of 1,000 patients with a median age of 56.00 ([IQR: 45.00-64.00) years, 56% of whom were male. The cohort comprised 500 CIM patients, with a median age of 57.50 (IQR: 48.00-65.00) years (53.8% male), and 500 non-CIM patients, with a median age of 53.50 (IQR: 42.00-63.00) years (58.2% male).The baseline characteristics of the experimental set are shown in **Supplementary Table S2**, and those of the external validation set are presented in **Supplementary Table S3**.

### Preliminary feature selection

Myelosuppression occurrence (present = 1, absent = 0) was used as the dependent variable, with 52 candidate factors as independent variables. This study selected the variables corresponding to lambda.1SE as the optimal penalty parameter, ultimately retaining 15 optimal features. The selection process is illustrated in **Figures 2A, B**. Fifteen biomarkers were selected from the training set using LASSO regression:white blood cell count (WBC, 4.0–10×10^9^/L), monocyte percentage (MONO%, 3.0%–10.0%), red blood cell count (RBC, 4.36–6.12×10^12^/L), mean corpuscular volume (MCV,75.0–98.3 fL), red blood cell distribution width–coefficient of variation (RDW-CV, 11.0%–16.0%), lymphocyte (LYMPH, 1.15–3.43), mean corpuscular hemoglobin concentration (MCHC, 313–348 g/L), eosinophil percentage (EOS,0.03-0.51×10^9^/L), high-density lipoprotein cholesterol (HDL-C, 1.0–1.5 mmol/L), alkaline phosphatase (ALP, 46–116 U/L), bicarbonate (HCO_3_^–^, 22.0–29.0 mmol/L), platelet count (PLT, 100–300×10^9^/L), mean platelet volume (MPV, 8.4–12.7 fL), plateletcrit (PCT, 0.169%–0.374%), and neutrophil percentage (NEUT%, 41.2%–73.5%).As shown in **Table 1**.

**Figure 2:**
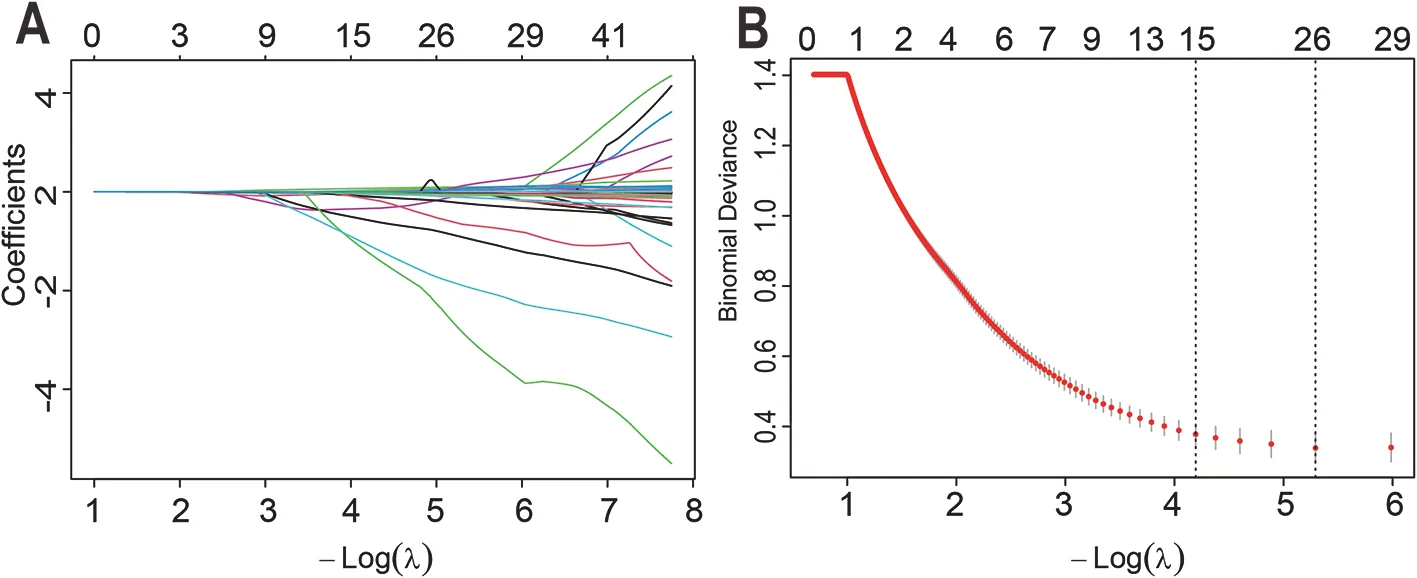
Shows the LASSO regression coefficient path and cross-validation curve.

**Table 1:** Baseline characteristics of the study cohort.

Variables	Total (N = 1000)	Non-CIM (n = 500)	CIM (n = 500)	*P value*
WBC,10^9/L, median[IQR]	4.65 (3.12,6.65)	6.13 (4.85,7.60)	3.22 (2.26,4.23)	<0.001^*^
MONO%,points, median[IQR]	6.60 (5.10,9.20)	5.90 (4.90,7.20)	8.45 (5.33,12.28)	<0.001^*^
RBC,10^12/L, median[IQR]	4.47 (3.89,4.99)	4.88 (4.46,5.40)	4.05 (3.47,4.49)	<0.001^*^
MCV,fL, median[IQR]	92.60 (89.10,96.70)	91.80 (88.60,94.90)	93.80 (89.60,99.00)	<0.001^*^
RDW-CV,points, median[IQR]	13.90 (13.00,15.40)	13.40 (12.90,14.30)	14.60 (13.30,16.30)	<0.001^*^
LYMPH,points, median[IQR]	1.20 (0.83,1.75)	1.59 (1.08,2.11)	0.98 (0.69,1.29)	<0.001^*^
MCHC,g/L, median[IQR]	330.00 (320.00,336.00)	333.00 (320.00,338.00)	322.00 (313.25,331.00)	<0.001^*^
EOS,points, median[IQR]	0.70 (0.03,0.13)	0.09 (0.05,0.18)	0.05 (0.02,0.10)	<0.001^*^
HDL-C,mmol/L, median[IQR]	1.07 (0.89,1.27)	1.11 (0.95,1.32)	1.02 (0.83,1.20)	<0.001^*^
ALP, U/L, median[IQR]	82.89 (66.23,103.46)	79.42 (64.18,96.00)	88.00 (69.00,113.49)	<0.001^*^
HCO3,mmol/L, median[IQR]	25.70 (24.20,27.20)	26.00 (24.23,27.58)	25.60 (24.13,27.00)	0.059
PLT,10^9/L, median[IQR]	191.00 (129.00,248.00)	213.00 (166.25,258.00)	155.00 (91.25,224.00)	<0.001^*^
MPV,fL, median[IQR]	9.20 (8.40,10.00)	9.40 (8.63,10.30)	8.80 (8.20,9.70)	<0.001^*^
PCT,points, median[IQR]	0.17 (0.12,0.23)	0.20 (0.16,0.24)	0.14 (0.08,0.20)	<0.001^*^
NEUT%,points, median[IQR]	60.40 (50.80,70.20)	63.80 (55.73,72.78)	56.30 (44.55,66.90)	<0.001^*^

Demographic and baseline characteristics of CIM and Non-CIM groups. CIM, chemotherapy caused bone marrow suppression; WBC, white blood cell count; MONO%, monocyte percentage; RBC, red blood cell count; MCV, mean corpuscular volume; RDW-CV, red blood cell distribution width–coefficient of variation; LYMPH, lymphocyte; MCHC, mean corpuscular hemoglobin concentration; EOS, eosinophil; HDL-C, high-density lipoprotein cholesterol; ALP, alkaline phosphatase; HCO_3_^–^, bicarbonate; PLT, platelet count; MPV, mean platelet volume; PCT, plateletcrit; NEUT%, neutrophil percentage; *P < 0.05.

### Performance and clinical applicability evaluation of the CIM prediction model

As shown in **Figure 3** and **Supplementary Table S4**, the evaluation results of the performance and clinical applicability for different ML models are presented. Based on the training set data, the LightGBM model demonstrated the best predictive performance, with an AUC of 0.999 (95% CI: 0.998–1.000) (**Figures 3A, C**), a sensitivity of 0.986(0.949-1.000), a specificity of 0.989 (0.951-1.000), an accuracy of 0.987 (0.962-1.000), a PPV of 0.989 (0.954-1.000), an NPV of 0.986 (0.963-1.000) , an F1-score of 0.987 (0.965-1.000) , and a Youden’s index of 0.974 (0.933-0.999) (**Supplementary Table S2**). When evaluated on the independent validation set, the LightGBM model also exhibited robust performance, achieving an AUC of 0.959 (95% CI: 0.939–0.979) (**Figures 3B, D**), a sensitivity of 0.933 (0.910-0.980), a specificity of 0.847 (0.783-0.897), an accuracy of 0.890(0.857-0.927), a PPV of 0.861 (0.803-0.907), an NPV of 0.941 (0.901-0.977), an F1-score of 0.902 (0.862-0.931), and a Youden’s index of 0.793 (0.719-0.855).

**Figure 3:**
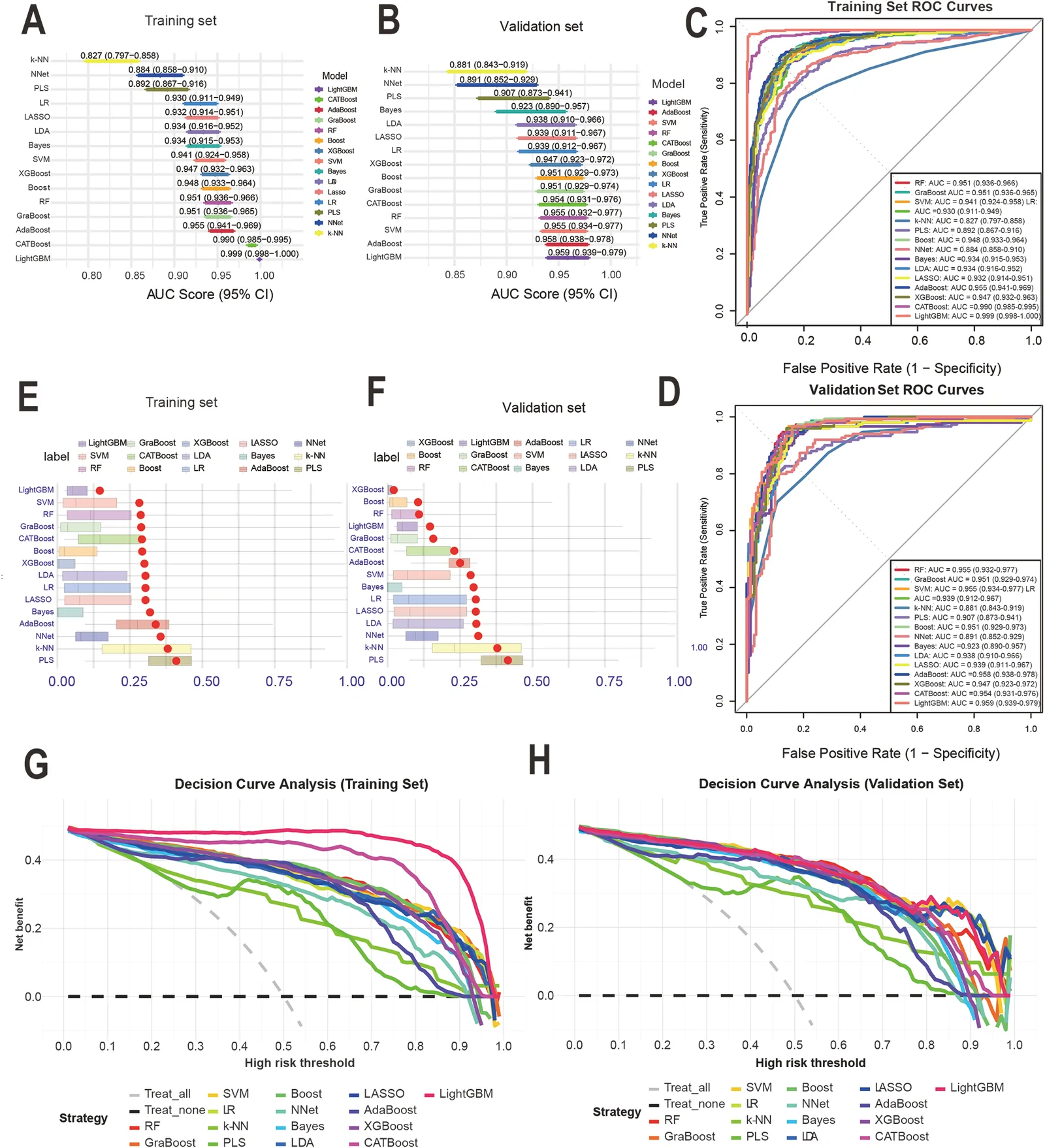
Performance evaluation of the ML for CIM prediction. **(A)** Forest plot for the AUC score of training set data; **(B)** Forest plot for the AUC score of validation set data; **(C)** ROC curve of training set data; **(D)** ROC curve of validation set data; **(E)** Absolute residual box plot of training set data; **(F)** Absolute residual box plot of validation set data; **(G)** DCA curve plot of training set data; **(H)** DCA curve plot of validation set data.

Notably, in the external validation cohort, the CatBoost model achieved slightly superior numerical metrics compared with the LightGBM model; however, the differences in AUC, accuracy, and clinical net benefit were marginal and not clinically meaningful.

In the boxplots of absolute residuals for the training set, the LightGBM model exhibited the most concentrated residual distribution, the least data fluctuation, and the lowest root mean square error, indicating the smallest overall prediction error for this model (**Figure 3E**). In the validation set, however, the XGBoost model showed the best performance according to this metric, while LightGBM ranked fourth among the 15 machine learning algorithms (**Figure 3F**).

The LightGBM model was preferred as the final model due to its excellent stability in both training and internal validation, faster computational speed, lower memory consumption, and better compatibility with high-dimensional routine laboratory data, which render it more suitable for clinical deployment and real-time risk calculation.

We employed DCA to evaluate the clinical applicability of the constructed ML models for CIM prediction. The results showed that, within a certain threshold probability range, the LightGBM model yielded a favorable net benefit similar to that of CatBoost, supporting its acceptable clinical applicability among the evaluated ML models.

### Internal validation of the LightGBM model for CIM prediction

The model validation results indicated that the optimal model (LightGBM) exhibited excellent classification performance on the training set. Its confusion matrix for the training set (**Figure 4A**) showed a high accuracy of 98.7%, with a low misclassification rate for both CIM and Non-CIM categories, including only 5 false negative and 4 false positive cases. The confusion matrix for the validation set (**Figure 4B**) demonstrated an accuracy of 89.0%, with misclassification rates of 10 false negatives and 23 false positives for the CIM and Non-CIM categories, respectively, indicating good recognition capability of the model.

**Figure 4:**
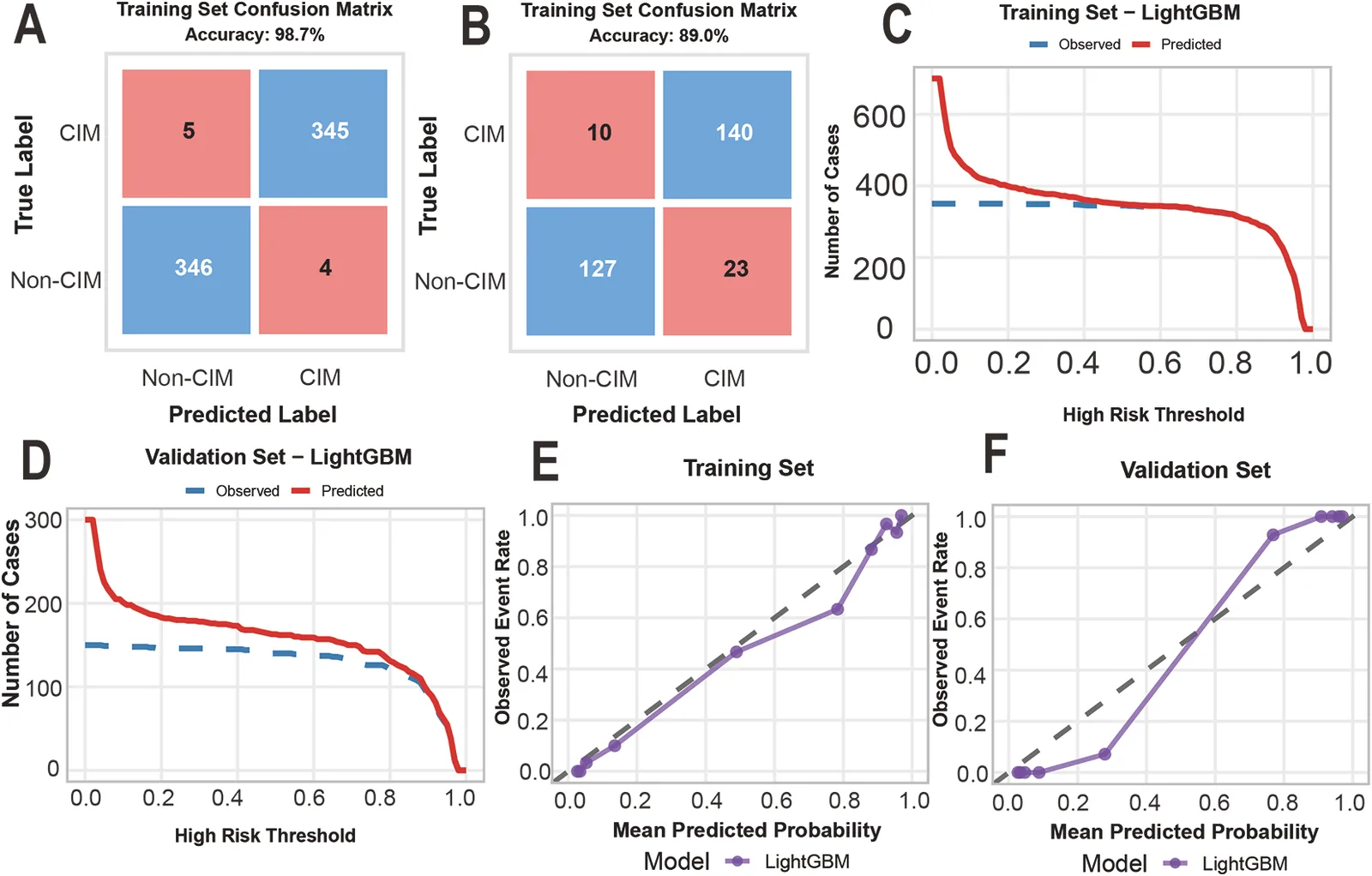
Validation of the LightGBM Model in CIM. **(A)** Confusion matrix of training set data **(B)** Confusion matrix of validation set data **(C)** Clinical impact curve of training set data **(D)** Clinical impact curve of validation set data **(E)** Calibration curve of training set data **(F)** Calibration curve of validation set data.

Further analysis of the risk threshold characteristics of the LightGBM model using the clinical impact curve revealed that, in both the training set (**Figure 4C**) and the validation set (**Figure 4D**), the number of predicted high-risk cases (red curve) showed a consistent decreasing trend as the risk threshold increased. Moreover, this trend closely aligned with the distribution of the actual observed cases (blue curve), suggesting robust discriminatory ability of the model for risk stratification. The calibration curves (**Figures 4E, F**) showed that, on both the training and validation sets, the mean predicted probability exhibited a good linear relationship with the actual event rate, closely approximating the ideal calibration line.

### SHAP visualization of the LightGBM model for CIM prediction

As shown in **Figure 5A**, SHAP values from the LightGBM model were used to determine the feature importance for predicting CIM. **Figure 5B** displays the SHAP summary plot: the x-axis represents the SHAP value, indicating the impact of a predictor on the model output. Different predictors are colored based on their feature values, with yellow representing high values and purple representing low values. A concentration of yellow points on the left side suggests a negative correlation (lower values are associated with higher CIM risk), while a concentration on the right side indicates a positive correlation (higher values are associated with higher CIM risk). The y-axis lists the features ranked by their overall importance.

**Figure 5:**
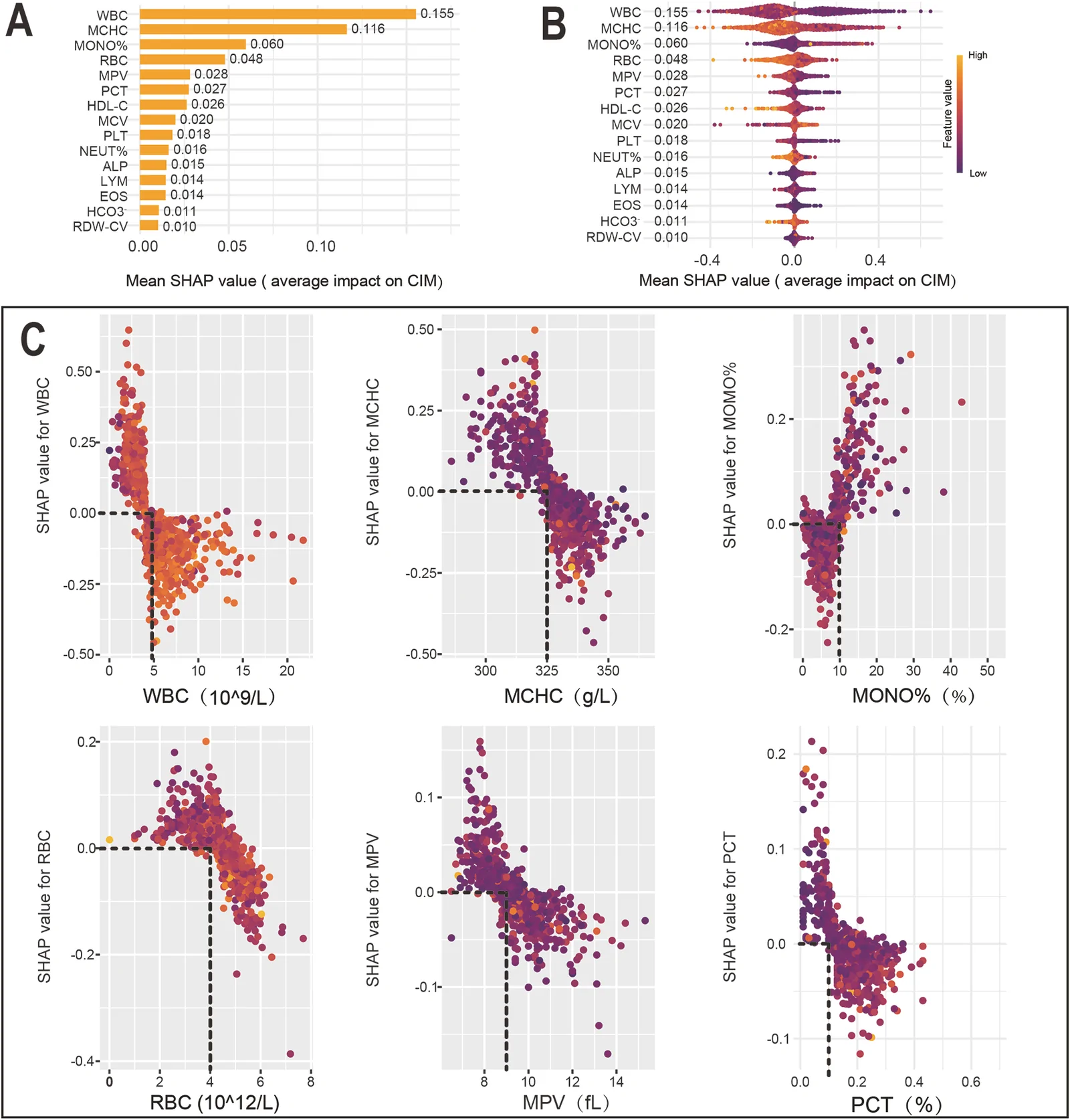
SHAP Visualization Analysis of the ML for CIM. **(A)** SHAP Summary bar plot **(B)** SHAP Beeswarm plot **(C)** SHAP dependence scatter plots for key features.

The results reveal that the risk of CIM development is significantly associated with the following predictors: WBC is the most significant predictor (showing a negative correlation), followed by MCHC, MONO%, RBC, MPV, and PCT, among others.

### Individualized feature attribution analysis of the LightGBM model for CIM prediction

Building upon the SHAP summary plot, the SHAP dependence plots for the top six most influential clinical features were generated to illustrate the specific impact of these predictors on the model’s output (**Figure 5C**). In these plots, the x-axis represents the range of the clinical feature, and the y-axis represents its corresponding SHAP value. A SHAP value > 0 indicates an increased predicted risk of myelosuppression for the patient. The color of the scatter points represents the actual value of an interacting feature.

The results demonstrate that WBC, MCHC, RBC, MPV, and PCT show a negative correlation with SHAP values, indicating that lower values of these features increase the predicted risk. Conversely, MONO% shows a positive correlation, where higher values increase the predicted risk. The SHAP dependence plots reveal that the risk of CIM increases when the following specific thresholds are met: WBC < 5 × 10^9^/L, MCHC < 325 g/L, MONO%> 10%, RBC < 4 × 10^12^/L, MPV < 8 fL, and PCT < 0.1%.

### External validation

The performance of the LightGBM model was comprehensively evaluated in an independent external validation cohort (**Figure 6**). In the external validation set, the ROC curve showed excellent discrimination, with an AUC of 0.940 (95% CI: 0.920–0.959), sensitivity of 0.818, specificity of 0.888, PPV of 0.882, NPV of 0.826, F1-score of 0.849, and Youden index of 0.706. The confusion matrix demonstrated an overall accuracy of 85.2%, with stable classification performance between predicted and observed groups, indicating high ability to distinguish outcome events from non-events. The calibration curve revealed that the mean predicted probabilities were highly consistent with the observed event rates, suggesting favorable model calibration and reliable risk estimation. DCA showed that the LightGBM model achieved a higher net benefit than the “treat-all” and “treat-none” strategies across a wide range of high-risk thresholds, confirming its favorable clinical utility. The external clinical impact curve clearly illustrated the stratification of case numbers by predicted risk, enabling effective identification of high-risk populations to guide clinical intervention.

**Figure 6:**
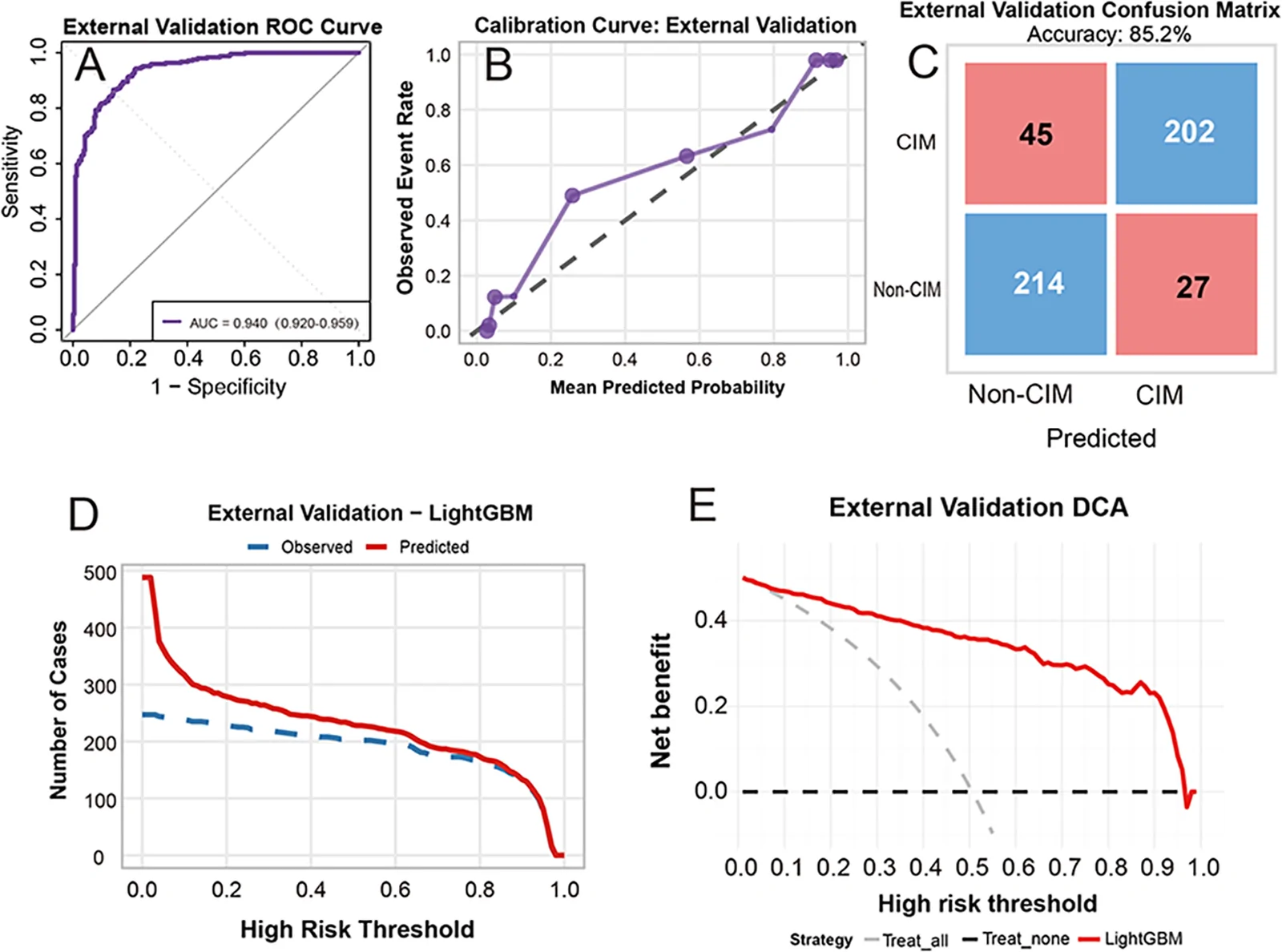
External Validation of the LightGBM Model in CIM. **(A)** ROC curve **(B)** Calibration curve **(C)** Confusion matrix **(D)** Clinical impact curve **(E)** DCA curve.

## Discussion

This study developed and validated 15 ML models that ultimately utilized 15 routine laboratory biomarkers to predict the risk of CIM in cancer patients. Among them, the LightGBM algorithm model demonstrated superior overall performance compared to other models, exhibiting high specificity, accuracy, and sensitivity in both the training and validation sets. The constructed LightGBM model showed excellent reliability and clinical applicability during internal validation. Furthermore, we provided SHAP dependence plots to facilitate rapid decision-making by medical professionals, which may contribute to early clinical intervention and more efficient allocation of healthcare resources.

In recent years, significant progress has been made in the research of clinical prediction models for CIM. However, traditional logistic regression analysis methods still exhibit notable limitations. While demonstrating strong generalizability, these methods commonly suffer from inherent drawbacks such as high false positive rates, multicollinearity, and model overfitting (18, 19). Furthermore, traditional statistical approaches are hampered by significant shortcomings, including a limited capacity for incorporating risk factors and constrained room for model optimization. Regarding data processing, traditional statistical methods have increasingly struggled to meet contemporary research demands. Although they can quickly identify differences between datasets and screen for predictive factors, their significance tests are overly sensitive to sample size and the number of variables, often leading to a discrepancy between statistical significance and clinical relevance (20). This is a key reason for inconsistent or even contradictory conclusions across different studies. More importantly, traditional methods face fundamental challenges when dealing with big data: they are ineffective in handling large-scale datasets, diverse data types, and complex variable interactions—common challenges in modern research. In contrast, machine learning algorithms demonstrate distinct advantages. This technology not only enhances the accuracy and performance of prediction models but also helps mitigate multicollinearity. Machine learning models are inherently prone to overfitting, which was reduced in this study using 10-fold cross-validation, hyperparameter optimization, and external validation. These strengths have led to its increasingly widespread application in the field of disease risk prediction (21).

Compared with conventional statistical methods such as logistic regression, the LightGBM algorithm possesses unique strengths for predicting chemotherapy-induced myelosuppression. Restricted by parametric linear logit assumptions, conventional regression models are incapable of autonomously detecting higher-order interactions among hematological biomarkers and can only test manually specified interaction terms. Even with LASSO pre-selection, penalty-driven sparsity may remove biologically meaningful but collinear predictors. As a histogram-based gradient boosting tree, LightGBM performs loss-guided node splitting free from parametric constraints, naturally capturing piece-wise and non-monotonic risk patterns and tolerating multicollinearity among correlated laboratory features. The risk thresholds observed in our analysis represent data-learnt breakpoints rather than arbitrarily predefined cut-offs.

The “black-box” nature of machine learning models often results in a lack of interpretability regarding their internal decision-making processes. To enhance the transparency of the model’s decisions, this study employed the SHAP method for a visual interpretability analysis of the LightGBM model. The results indicated that the top six risk factors with the greatest influence on the model’s output were WBC, MCHC, MONO%, RBC, MPV, and PCT. Previous studies have shown that a low pre-chemotherapy white blood cell count is an independent risk factor for CIM (22, 23), which aligns with the findings of this research. As a sensitive indicator of bone marrow hematopoietic function, white blood cell count holds a primary position in predicting CIM. Its predictive value stems from the extreme sensitivity of white blood cell populations, particularly neutrophils, to cytotoxic drugs. Chemotherapeutic agents not only directly kill proliferating hematopoietic precursor cells but also indirectly suppress hematopoietic function by damaging stromal cells within the bone marrow microenvironment. When a patient’s pre-chemotherapy WBC level is low (<4.0×10^9^/L), it indicates that their bone marrow reserve function is already at a compensatory limit and cannot withstand subsequent chemotherapy.However, this interpretation requires clinical caution, as several benign conditions may also lead to low WBC without increasing CIM risk, including Duffy-null genotype–related benign neutropenia,transient viral infection,which should be carefully distinguished in clinical practice.

MCHC is closely associated with bone marrow erythropoietic activity and serves as a key marker of hematopoietic resilience. Low MCHC (<320 g/L) indicates impaired iron utilization and mitochondrial dysfunction in hematopoietic stem cells, which compromises bone marrow reserve and red blood cell production efficiency, thereby increasing susceptibility to chemotherapy-induced oxidative stress and myelosuppression (24). Chemotherapy damages rapidly proliferating bone marrow hematopoietic stem and progenitor cells, triggering hematopoietic stress and sterile inflammatory responses in the bone marrow. To maintain innate immunity, the body initiates emergency hematopoietic compensation, which promotes the biased differentiation of hematopoietic stem and progenitor cells toward the MONO lineage and accelerates the release of MONO into the peripheral blood. Meanwhile, neutrophils are more sensitive to chemotherapy due to their rapid proliferation, high turnover rate and short half-life, and undergo massive apoptosis with a significant decrease in number during the early stage of CIM. The increased release, longer survival and milder reduction of MONO, combined with the rapid decrease of neutrophils, collectively result in a relative elevation of MONO% in the peripheral blood. This change occurs earlier than the significant decrease of neutrophils and can therefore be used as an early warning indicator for impending CIM (25).

RBC count directly reflects bone marrow erythroid pool activity and hematopoietic stem cell homeostasis. Low RBC (<3.5×10^12^/L) indicates impaired erythroid differentiation, which also compromises granulocytic and megakaryocytic function, increasing the risk of chemotherapy-related pancytopenia (26). MPV is a sensitive indicator of megakaryocyte maturation and bone marrow hematopoietic activity. Low MPV suggests insufficient bone marrow reserve, impaired platelet production, and poor recovery potential, which exacerbate chemotherapy-induced thrombocytopenia and form a vicious cycle of megakaryocyte dysfunction (27). PCT reflects total platelet mass and is more sensitive than platelet count alone for evaluating megakaryocyte function. Low PCT (<0.15%) indicates impaired platelet production or accelerated destruction, and may serve as an early marker of bone marrow damage (28).

The risk thresholds determined by SHAP analysis (WBC <5×10^9^/L, MONO% >10%, MCHC <325 g/L, RBC <4×10^12^/L, MPV <8 fL, PCT <0.1%) are generally consistent with routine clinical reference ranges and align with the early warning principles recommended in the Expert Consensus on the Out-of-Hospital Management of Tumor Therapy-Related Myelosuppression (2025 Edition).Specifically, WBC <5×10^9^/L represents a commonly recognized cutoff for insufficient bone marrow reserve; MONO% >10% reflects early bone marrow stress and relative hematopoietic compensation; MCHC <325 g/L indicates impaired erythropoietic function and reduced hematopoietic stability; RBC <4×10^12^/L suggests decreased erythroid marrow activity; MPV <8 fL reflects weakened megakaryocyte maturation and low platelet production capacity; and PCT <0.1% indicates reduced total platelet mass and insufficient marrow reserve.Baseline laboratory data are obtained within 24 hours before the first cycle of chemotherapy. The 15 features selected by LASSO regression are used as model input to generate an individual probability of CIM.The model can be deployed as a simple standalone calculator or integrated into the electronic health record (EHR) as a clinical decision support tool. Risk probabilities can be computed automatically once laboratory data are available.It assists clinicians in rapidly identifying high-risk populations, Although the risk ranges derived from this model are generally consistent with the warning principles in published expert consensus documents, prophylactic interventions should not be regarded as direct outputs or automatic consequences of the model. All preventive or therapeutic measures must be implemented within a standardized clinical decision framework and in accordance with current clinical guidelines after comprehensive physician-led assessment of individual patient conditions.

Although this study achieved promising performance in the early prediction of CIM, several limitations remain. Significant differences existed in baseline characteristics across groups, which may introduce residual confounding and compromise model stability. The 1:1 case-control sampling adopted in this study was an active design rather than naturally formed during patient recruitment. In routine clinical settings, CIM represents an imbalanced outcome, with its incidence far lower than 50% among consecutively enrolled patients. If a model is directly trained on such imbalanced datasets, it tends to be biased toward the majority class, resulting in suboptimal sensitivity for identifying patients with true CIM. Balancing the development cohort via case-control matching, class weight adjustment, or resampling is a widely accepted strategy in clinical machine learning prediction research. This approach compels the algorithm to learn discriminative patterns between features and outcomes, instead of merely memorizing prior class probabilities. Moreover, equal sample sizes for event and non-event groups in the training set ensure sufficient CIM cases within each fold of cross-validation, stabilizing hyperparameter tuning, reducing variability in feature selection, and providing adequate statistical power for subsequent SHAP-based subgroup interpretability analyses.

However, the artificially set 50% CIM event rate in the development cohort may lead to biased estimates of PPV, NPV, individual absolute predicted probabilities, calibration metrics, and net benefit in DCA. Accordingly, the risk probabilities output by the LightGBM model constructed from the dataset of this study should be regarded as relative risk scores for patient stratification, rather than accurate absolute CIM probabilities in the real world, and cannot be directly used to inform patients of their absolute clinical risk. The independent external validation cohort of this study consisted of consecutively enrolled patients without artificial case-control matching and yielded acceptable calibration and discrimination. Nevertheless, external validation cannot fully eliminate bias introduced by balanced sampling during model training. Further recalibration and performance reassessment of the model in prospective cohorts with real-world CIM incidence are required to verify the reliability of absolute risk prediction. Despite the external validation performed in this study, multicenter prospective studies are still needed to validate the stability and generalizability of the model across diverse populations and chemotherapy regimens.

## Conclusion

The LightGBM model developed in this study demonstrates good discrimination and calibration, offering reliable predictive performance. Healthcare professionals can utilize routine laboratory biomarkers to assess the risk of chemotherapy-induced myelosuppression, thereby strengthening risk management during chemotherapy for cancer patients, implementing targeted interventions, reducing chemotherapy-related adverse outcomes, and ultimately alleviating the economic burden on patients while improving prognosis. Furthermore, by applying SHAP-based interpretability analysis to the LightGBM model, this study enhances the transparency and reliability of the model’s decision-making process, aiding clinical staff in understanding the prediction logic and supporting clinical decision-making. Future work will involve multicenter clinical studies to validate the model’s stability across diverse populations and treatment regimens, as well as prospective cohort studies to explore its value for early identification and intervention of CIM in resource-limited settings.

## Data Availability

The original contributions presented in the study are included in the article/**Supplementary Material**. Further inquiries can be directed to the corresponding authors.

## References

[1] Sung H Ferlay J Siegel RL Laversanne M Soerjomataram I Jemal A . Global cancer statistics 2020: GLOBOCAN estimates of incidence and mortality worldwide for 36 cancers in 185 countries. CA Cancer J Clin. (2021) 71:209–49. doi: 10.3322/caac.21660 33538338

[2] Maomao C He L Dianqin S Siyi H Xinxin Y Fan Y . Current cancer burden in China: epidemiology, etiology, and prevention. Cancer Biol Med. (2022) 19:1121–38. doi: 10.20892/j.issn.2095-3941.2022.0231 36069534 PMC9425189

[3] Podina R Todea D Fodor LA . Fear of cancer recurrence and mental health: a comprehensive meta analysis. Psycho-oncology. (2023) 32:1503–13. doi: 10.1002/pon.6205 37596855

[4] Wang HQ Lin H Liu B . Research progress on the psychological burden and intervention measures in cancer patients. Front Psychiatry. (2024) 15:1408762. doi: 10.3389/fpsyt.2024.1408762 38938456 PMC11208330

[5] Fuentes-Alabi S . Navigating the economic challenges in childhood cancer control in low- and middle-income countries: insights from the CC-BRIDGE tool and the global initiative for childhood cancer. Cancer. (2024) 130:1025–7. doi: 10.1002/cncr.35209 38240557

[6] Vaghari-Tabari M Jafari-Gharabaghlou D Mohammadi M Hashemzadeh MS . Zinc oxide nanoparticles and cancer chemotherapy: helpful tools for enhancing chemo-sensitivity and reducing side effects? Biol Trace Elem Res. (2024) 202:1878–900. doi: 10.1007/s12011-023-03803-z 37639166

[7] Crawford J Herndon D Gmitter K Weiss J . The impact of myelosuppression on quality of life of patients treated with chemotherapy. Future Oncol. (2024) 20:1515–30. doi: 10.2217/fon-2023-0513 38587388 PMC11441072

[8] Nian Q Liu RX Zeng JH . Unraveling the pathogenesis of myelosuppression and therapeutic potential of natural products. Phytomedicine. (2024) 132:155810. doi: 10.1016/j.phymed.2024.155810 38905848

[9] Boccia R Glaspy J Crawford J Aapro M . Chemotherapy induced neutropenia and febrile neutropenia in the US: a beast of burden that needs to be tamed? Oncologist. (2022) 27:625–36. doi: 10.1093/oncolo/oyac074 35552754 PMC9355811

[10] Huang R Li T Li MX Ye ST Nie N Liang Y . Systematic review and Meta-analysis on chemotherapy-induced bone marrow suppression treated with acupuncture. Zhong guo Zhen Jiu. (2021) 41:557–62. doi: 10.13703/j.0255-2930.20200618-k0005 34002574

[11] Weycker D Hatfield M Grossman A Hanau A Lonshteyn A Sharma A . Risk and consequences of chemotherapy-induced thrombocytopenia in US clinical practice. BMC Cancer. (2019) 19:151–5. doi: 10.1186/s12885-019-5354-5 30764783 PMC6376753

[12] Mones JV Soff G . Management of thrombocytopenia in cancer patients. Cancer Treat Res. (2019) 179:139–50. doi: 10.1007/978-3-030-20315-3_9 31317485

[13] Zhang M Zheng Y Maidaiti X Liang B Wei Y Sun F . Integrating machine learning into statistical methods in disease risk prediction modeling: A systematic review. Health Data Sci. (2024) 4:165–70. doi: 10.34133/hds.0165 39050273 PMC11266123

[14] Wang X Zhang R Xie H Xu J Chu J Wei C . Risk factors analysis and nomogram development for myelosuppression in diffuse large B-cell lymphoma patients undergoing first-line chemotherapy: a dual-centre retrospective cohort study. PeerJ. (2025) 13:e19539. doi: 10.7717/peerj.19539 40547311 PMC12180452

[15] Ou H Tan Z Shen A Yu L He Y Hong R . Development of a machine learning-based risk prediction model and analysis of risk factors for docetaxel-induced bone marrow suppression in breast cancer patients. Int J Clin Pharm-Net. (2025) 47:1874–84. doi: 10.1007/s11096-025-01989-x 40856959

[16] Huang Y Mao J Li X . Emerging biomarkers in prostate cancer diagnosis and treatment: Insights into genetic, RNA and metabolic markers (Review). Int J Oncol. (2025) 68:1–17. doi: 10.3892/ijo.2025.5828 41347816 PMC12716904

[17] China Anti-Cancer Association Tumor Clinical Chemotherapy Professional Committee . Expert consensus on the out-of-hospital management of myelosuppression associated with oncological treatment (2025 edition). Zhonghua Yi Xue Za Zhi. (2025) 105:793–804. doi: 10.3760/cma.j.cn112137-20241029-02425 40091234

[18] Chen WQ Peng L Zeng XL Wen WP Sun W . Predictors of myelosuppression for patients with head and neck squamous cell carcinoma after induction chemotherapy. Clin Med Insights Oncol. (2024) 18:1–12. doi: 10.1177/11795549231219497 38187457 PMC10771758

[19] Xie H Zhang R Wei C Xu J Chu J Wang X . Construction and validation of a nomogram prediction model for predicting the risk of chemotherapy-induced myelosuppression after chemotherapy in patients with triple-negative breast cancer: a single-center retrospective case-control study. Transl Cancer Res. (2025) 14:2885–99. doi: 10.21037/tcr-24-1513 40530156 PMC12170043

[20] Rambaran S Naidoo K Lewis L Hassan-Moosa R Govender D Samsunder N . Effect of inflammatory cytokines/chemokines on pulmonary tuberculosis culture conversion and disease severity in HIV-infected and -uninfected individuals from south africa. Front Immunol. (2021) 12:641065. doi: 10.3389/fimmu.2021.641065 33868272 PMC8047115

[21] Zhou Q Zuley M Guo Y Yang L Nair B Vargo A . A machine and human reader study on AI diagnosis model safety under attacks of adversarial images. Nat Commun. (2021) 12:72–81. doi: 10.1038/s41467-021-27577-x 34907229 PMC8671500

[22] Li M Wang Q Lu P Zhang D Hua Y Liu F . Development of a machine learning-based prediction model for chemotherapy-induced myelosuppression in children with wilms' Tumor. Cancers (Basel). (2023) 15:1078–81. doi: 10.3390/cancers15041078 36831423 PMC9954251

[23] Lyman GH Kuderer NM Crawford J Wolff DA Culakova E Poniewierski MS . Predicting individual risk of neutropenic complications in patients receiving cancer chemotherapy. Cancer. (2011) 117:1917–27. doi: 10.1002/cncr.25691 21509769 PMC3640637

[24] Mayhew CN Phillips JD Greenberg RN Birch NJ Elford HL Gallicchio VS . *In vivo* and *in vitro* comparison of the short-term hematopoietic toxicity between hydroxyurea and trimidox or didox, novel ribonucleotide reductase inhibitors with potential anti-HIV-1 activity. Stem Cells. (1999) 17:345–56. doi: 10.1002/stem.170345 10606163

[25] Ouyang W Liu Y Deng D Zhou F Xie C . The change in peripheral blood monocyte count: A predictor to make the management of chemotherapy-induced neutropenia. J Cancer Res Ther. (2018) 14:S565–70. doi: 10.4103/0973-1482.177502 30249869

[26] Gao A Zhang L Zhong D . Chemotherapy-induced thrombocytopenia: literature review. Discov Oncol. (2023) 14:10–20. doi: 10.1007/s12672-023-00616-3 36695938 PMC9877263

[27] Xiao H Xiong L Song X Jin P Chen L Chen X . Angelica sinensis Polysaccharides Ameliorate Stress-Induced Premature Senescence of Hematopoietic Cell via Protecting Bone Marrow Stromal Cells from Oxidative Injuries Caused by 5-Fluorouracil. Int J Mol Sci. (2017) 18:2265–70. doi: 10.3390/ijms18112265 29143796 PMC5713235

[28] Bessman D . Prediction of platelet production during chemotherapy of acute leukemia. Am J Hematol. (1982) 13:219–27. doi: 10.1002/ajh.2830130305 7180835

